# Impact of population ageing on the costs of hospitalisations for cardiovascular disease: a population-based data linkage study

**DOI:** 10.1186/s12913-014-0554-9

**Published:** 2014-11-13

**Authors:** Ninh Thi Ha, Delia Hendrie, Rachael Moorin

**Affiliations:** Department of Community Health, Institute of Public Health at Ho Chi Minh City, 159 Hung Phu street, District 8, Ho Chi Minh City, Vietnam; School of Public Health, Curtin University, Bentley, Perth, Western Australia; Faculty of Health Science, Curtin University, Bentley, Perth, Western Australia

**Keywords:** Cardiovascular disease, Health expenditures, Population ageing, Data linkage

## Abstract

**Background:**

Cardiovascular disease (CVD) is the most costly disease in Australia. Measuring the impact of ageing on its costs is needed for planning future healthcare budget. The aim of this study was to measure the impact of changes in population age structure in Western Australia (WA) on the costs of hospitalisation for CVD.

**Methods:**

All hospitalisation records for CVD occurring in WA in 1993/94 and 2003/04 inclusive were extracted from the WA Hospital Morbidity Data System (HMDS) via the WA Data Linkage System. Inflation adjusted hospitalisation costs using 2012 as the base year was assigned to all episodes of care using Australian Refined Diagnosis Related Group (AR-DRG) costing information. The component decomposition method was used to measure the contribution of ageing and other factors to the increase of hospitalisation costs for CVD.

**Results:**

Between 1993/94 and 2003/04, population ageing contributed 23% and 30% respectively of the increase in CVD hospitalisation costs for men and women. The impact of ageing on hospitalisation costs was far greater for chronic conditions than acute coronary syndrome (ACS) and stroke.

**Conclusions:**

Given the impact of ageing on hospitalisation costs, and the disparity between chronic and acute conditions, disease-specific factors should be considered in planning for future healthcare expenditure.

**Electronic supplementary material:**

The online version of this article (doi:10.1186/s12913-014-0554-9) contains supplementary material, which is available to authorized users.

## Background

The population in developed countries is ageing as a result of the increase in life expectancy and low fertility [1]. It is generally agreed that these ageing populations will cause a considerable socioeconomic impact for the community as a whole and healthcare systems in particular [2]. However, an explanation for the link between healthcare expenditure and ageing is still a ‘black box’ [1]. To guide health policy, it is important to examine closely the influence of ageing on healthcare expenditure within a particular country.

In Australia, the proportion of those aged 65 years and older has increased from 11.1% in 1990 to 13.6% in 2010 [3]. It is projected that the proportion will increase to 16% by 2016 and 25% by 2051 [2]. Simultaneously, over the past decade, healthcare expenditure has increased at a rate higher than that of other goods and services [4]. The age-expenditure profile shows that higher costs are spent on the older population, for example, expenditure on healthcare for those aged 65 years and older was four times higher than those under 65 years [2,5]. Coupled with this, the life span living with disability has also been found to be increasing in many studies [6,7]. Due to this expansion in morbidity, the demand for healthcare services is likely to increase, in turn escalating healthcare expenditure [2,8].

While the relationship between ageing and healthcare expenditure in Australian and other developed countries has been examined in the literature [1,5,9-12], there is a discordance regarding the magnitude of the impact of ageing on healthcare expenditure. For instance, studies in France have found that only 3.4% of the rise in healthcare expenditure was attributable to ageing while in Korea it was approximately 10% [9,13]. In contrast, a study in Australia projected that the ageing population would contribute to almost 50% of the increase in healthcare expenditure between 2002/03 and 2044/45 [5]. Although those studies examined the impact of ageing on healthcare expenditure, each study were focused on different type of expenditure. The study in France were looked at a combination of ambulatory, pharmaceutical and hospitalisation costs while costs of health insurance in Korean, and government healthcare expenditure in Australia were examined. Country specific factors may be the cause of differences in the impact of ageing on healthcare expenditure [14,15].

Currently, cardiovascular disease (CVD) is a leading cause of death in Australia and the most costly disease, accounting for 11% of total healthcare expenditure in 2005/06 [16], with hospital services comprising the largest component of CVD total expenditure [17]. Ageing is an unmodifiable risk factor of all disease, including CVD. Thus, the ageing population may cause a considerable burden for costs of CVD. However, there is limited literature about the impact of the ageing population on the costs of CVD-related hospitalisation. Considering the rise in healthcare costs and the relative burden of CVD, an understanding of the magnitude of the impact of ageing on costs of treatment for CVD in Western Australia (WA) will provide a useful evidence base for planning and budgetary purposes and future policy formulation. The aim of the study was to measure the contribution of population ageing to the hospitalisation costs for CVD in WA, using the component decomposition method. Through the method, the hospitalisation costs were decomposed into subcomponents and subsequently costs attributable to ageing of the population in each component over a period of time were isolated.

## Methods

### Study population and data source

All hospitalisations occurring in Western Australia pertaining to patients aged 18 years and older with a principal or co-diagnosis CVD code from 1 July 1993 to 30 June 2004 were extracted from the Hospital Morbidity Data System (HMDS) using the Western Australian Data Linkage System (WADLS) described elsewhere [18,19]. The HMDC captures records of separations from all public, private acute care and psychiatric hospitals and private freestanding day hospital facilities in Western Australia [20]. The relevant CVD codes of hospitalisations were identified using the International Classification of Disease (ICD) codes version current at the time of separation from hospital and are detailed in the following sub section. Prior to data extraction, ethics approval for this study was obtained from the Human Research Ethics Committee at the University of Western Australia and Curtin University.

### Case selection and classification

Common types of CVD in Australia were included in this study. Relevant ICD codes to identify hospitalisations with CVD were obtained from publications produced by the Australian Institute of Health and Welfare (AIHW [17] and L Nedkoff, TG Briffa, DB Preen, FM Sanfilippo, J Hung, SC Ridout, M Knuiman and M Hobbs [21]. Events were defined as CVD when they were recorded as either heart failure, or angina pectoris, or acute myocardial infarction, or unstable angina or stroke. All records were subsequently grouped into three subgroups, namely chronic conditions (ICD 9_CM 428, and 413, ICD 10-AM I50, and I20), acute coronary syndromes (ACS) (ICD 9_CM 410, and 411.1, ICD 10-AM I21, and I20.0) and stroke (ICD 9_CM 430–432 and 436, ICD 10-AM I60-I64).

The individual records of hospitalisation were aggregated into episodes of care, defined as a period of continuous hospitalisation. Inter-hospital transfers were periods of in-patient hospitalisation that overlapped with each other or where separation (discharge) and admission dates were on sequential days. Capture of transfers between hospitals is required in order to avoid over counting of the number of hospitalisation events. In our study periods of contiguous in-patient hospitalisation were classified as episodes of care. The episode of care was assigned to one of 17 age groups (18–19, 20–24 to 90–94 in five-year age groups and 95+ years) determined by the age in years at admission time of each individual. The first age group comprised only two years (18–19 years) since data extraction was restricted to hospitalisation records for individuals from 18 years and over.

### Assigning cost to episodes of care

The Australian Refined Diagnosis Related Group is an Australian admitted patient classification system, which provides a way of relating the number and type of patients treated in a hospital to the resources required by the hospital. Acute admitted patient episodes of care are categorised into groups with similar conditions and similar usage of hospital resources, using information in the hospital morbidity record such as the diagnoses, procedures and demographic characteristics of patient.

The cost of each episode of care was assigned based on AR-DRG code recorded in the HMDS record for each record of hospitalisation included within an episode of care (where there were inter-hospital transfers). The cost attributed to each AR-DRG was that reported for that AR-DRG in the National Hospital Cost Data Collection Report relevant to the year of each separation [22]. The cost was assigned inclusive for all hospitalisation events in database before adjusting for the inter-hospital transfers and therefore included multiple records in the case of inter-hospital transfer. Thus, the costs of the inter-hospital transfer episodes were a sum of any records which formed the episode of care. The hospitalisation costs included medications, investigation undertaken whole admitted, physicians, nursing staff, operating theatre, emergency department and “hotel” costs but not included rehabilitation costs. All dollar values were adjusted to 2012 price levels, using health price indices calculated from the Health and Welfare expenditure series of the AIHW [23,24].

### Statistical analysis

The total number of episodes (E) and cost per episode (CPE) were calculated in five-year age groups by sex and diagnosis subgroup at two points of time, 1993/94 and 2003/04. Number of episodes per capita (NEC) for gender *x* and age-specific group *j* was calculated separately for chronic conditions, ACS, stroke and overall CVD:

$NE{C}_{xj}=\frac{Ex,j}{Px,j}$, where *Ex,j* is the number episodes for gender *x* and age-specific group *j*; *Px,j* is the WA population in gender x and age group j for that year (obtained from the Australian Bureau of Statistic Time Series Workbook Table 55 (ABS 3101.0)) as the denominator [25]. The rates were calculated per 100,000 population.

The cost per episode (CPE) for gender x and age specific group j was calculated for chronic conditions, ACS, stroke and overall CVD as following: $\mathrm{C}\mathrm{P}\mathrm{E}=\frac{Cx,j}{Ex,j}$, where *Cx,j* is total cost in gender *x* and age group *j*; *Ex,j* is the number of episodes in gender *x* and age group *j*.

In our study, the impact of population ageing on the hospitalisation costs for CVD was measured using the method that was adapted from the principles of the component decomposition method used in studies in Australia and Korea [12,13]. The previous studies, the change in healthcare expenditure was attributable to the impact of ageing, the growth of population over time, change in proportion of people using healthcare services and change in average cost per episode [12,13]. Similar to the studies, the change in total costs of hospitalisation for CVD over a period of time were attributable to following components: population growth, ageing of the population, the increase in total number of episodes of hospitalisations and the increase in average cost per episode. Steps to calculate proportion of contribution of each component to the change in total costs of hospitalisation for CVD between 1993/94 and 2004/05 were described in detail below.

The total costs of hospitalisations for disease *d* in each year was decomposed by the following equation:

1 $$ \begin{array}{l} Total\  hospitalisation\  cost{s}_d = \\ {}\sum\ WA\  populatio{n}_j*\  number\  of\  episod e\  per\  capit{a}_{d,j}*\ \\ {} cost\  per\  episod{e}_{d,j}; where\ j\  denotes\  five- year\  age\  groups\end{array} $$

From equation (1), the change in the WA population over the study period can be decomposed into a change in the total population and a change in the age distribution of population. Similarly, the change in the number of hospital episodes per capita can be decomposed into a change in the total number of episodes and a change in the age distribution of episodes. Thus, a difference in the total hospitalisation costs between 1993/94 and 2003/04 (DHC) was attributable to the change in total population (CPOP), the change in the age distribution of population (CDEM1), the change in the total number of episodes (CTNE), the change in the age distribution of episodes (CDEM2) and a change in cost per episode (CCPE).

A change in total hospitalisation costs for a disease *d* for the time period *t* (1993/93 and 2003/04) was

$$ \mathrm{D}\mathrm{H}{\mathrm{C}}_{\mathrm{d}} = \mathrm{C}\mathrm{P}\mathrm{O}{\mathrm{P}}_{\mathrm{d}} + \mathrm{C}\mathrm{DEM}{1}_{\mathrm{d}} + \mathrm{C}\mathrm{DEM}{2}_{\mathrm{d}} + \mathrm{C}\mathrm{T}\mathrm{N}{\mathrm{E}}_{\mathrm{d}} + \mathrm{C}\mathrm{C}\mathrm{P}{\mathrm{E}}_{\mathrm{d}} $$

The total of changes attributable to the change in the age structure of the population (CDEM1_*d*_) and to the distribution of hospitalisation between age groups CDEM2_*d*_ reflect the impact of ageing on total costs, thus impact of ageing = CDEM1_*d*_ + CDEM2_*d*_. Through this analysis the impact of the ageing of the population on the cost of hospitalisation for CVD was isolated from the respective costs of the increase in the general population and the total number of hospitalisations.

In order to calculate the proportion of contribution of each component, an age-specific cost profile, represents the per capita healthcare cost of a specific age group, was applied [26]. By holding age-specific cost profiles constant, this approach has an assumption that the impact of other variables with potential influence on healthcare cost does not change [26]. Although the assumption is unrealistic, it is useful for the purpose of isolating the impact of demographic changes (ageing population). The method has been used in many studies [12,26,27].

In our study, to capture the proportion of each component contributing to the difference in total cost of hospitalisation in the period, each component in initial year (1993/94) was moved to actual value in 2003/04 in sequence. As each component in the assumption was changed, the difference in total cost of hospitalisation between initial year (1993/94) and the assumed final year (2003/04) was attributable to whichever component was affected by the change in assumption. Each assumption using the actual value in 2003/04, the sum of the change in individual components is equal to the change in total cost between the two points of time. Details of the calculations are presented in Additional file 1.

While it is common practice that sensitivity analysis is conducted on health economic analyses to evaluate the effect of various assumptions and the averaging of values have on the magnitude of the final outcome, in this study there were no assumptions made since the data were not a sample and the number of hospitalisations and costs were those recorded directly in the data set. Thus there were no values that could be varied for use in a sensitivity analysis. Descriptive analysis was conducted using SPSS version 18. Calculations for the decomposition analysis were done in Microsoft Excel 2010.

## Results

The dataset consisted of 25,126 episodes of care for any discharged diagnosis with CVD reported in the WADLS in 1993/94 and 2003/04, of which almost 90% were for chronic conditions and ACS and only 11.7% for stroke (Table 1). Patients admitted for chronic conditions tended to be older, and those admitted with ACS younger, than the mean age across all CVD patients of 70.3 years (SD 12.9 years) in 1993/94 and to 69.7 years (SD 13.8 years) in 2003/04. The proportion of men was higher than women in all hospitalisations for CVD and subgroups. Compared with 1993/94, the number of episode per 100,000 population and mean cost per episode in 2003/04 were higher for CVD and all subgroups (Table 1).

**Table 1:** **Characteristics of hospitalisations for cardiovascular disease and subgroups**

	**CVD**	**Chronic conditions**	**ACS**	**Stroke**
**Total episodes**	25,126 (100.0)	11,334 (45.1)	10,845 (43.5)	2,947 (11.7)
1993/94	10,592 (100.0)	4,645 (43.9)	4,669 (44.1)	1,278 (12.1)
2003/04	14,534 (100.0)	6,689 (46.0)	6,176 (42.5)	1,669 (11.5)
**Age (mean (SD))**				
1993/94	70.3 (12.9)	73.2 (12.5)	67.4 (12.9)	69.9 (12.2)
2003/04	69.7 (13.8)	71.3 (13.4)	67.8 (13.6)	70.5 (15.2)
**Sex**				
1993/94				
Men	6,064 (57.3)	2,354 (50.7)	2,903 (62.2)	807 (63.1)
Women	4,528 (42.7)	2,291 (49.3)	1,766 (37.8)	471 (36.9)
2003/04				
Men	8,760 (60.3)	3,991 (59.7)	3,927 (63.6)	842 (50.4)
Women	5,774 (39.7)	2,698 (40.3)	2,249 (36.4)	827 (49.6)
**Number of episode per capita (per 100,000)**				
1993/94	852.6	373.9	375.8	102.9
2003/04	972.4	447.5	413.2	111.7
**Mean cost per episode (A$)**				
1993/94	6,013.5 (4,134.4)	5,280.7 (2,441.1)	6,482.8 (5,126.7)	6,962.8 (4,512.5)
2003/04	8,040.3 (8,957.9)	6,428.8 (7,002.4)	8,548.1 (8,276.1)	12,619.4 (14,718.5)

**Figure 1:**
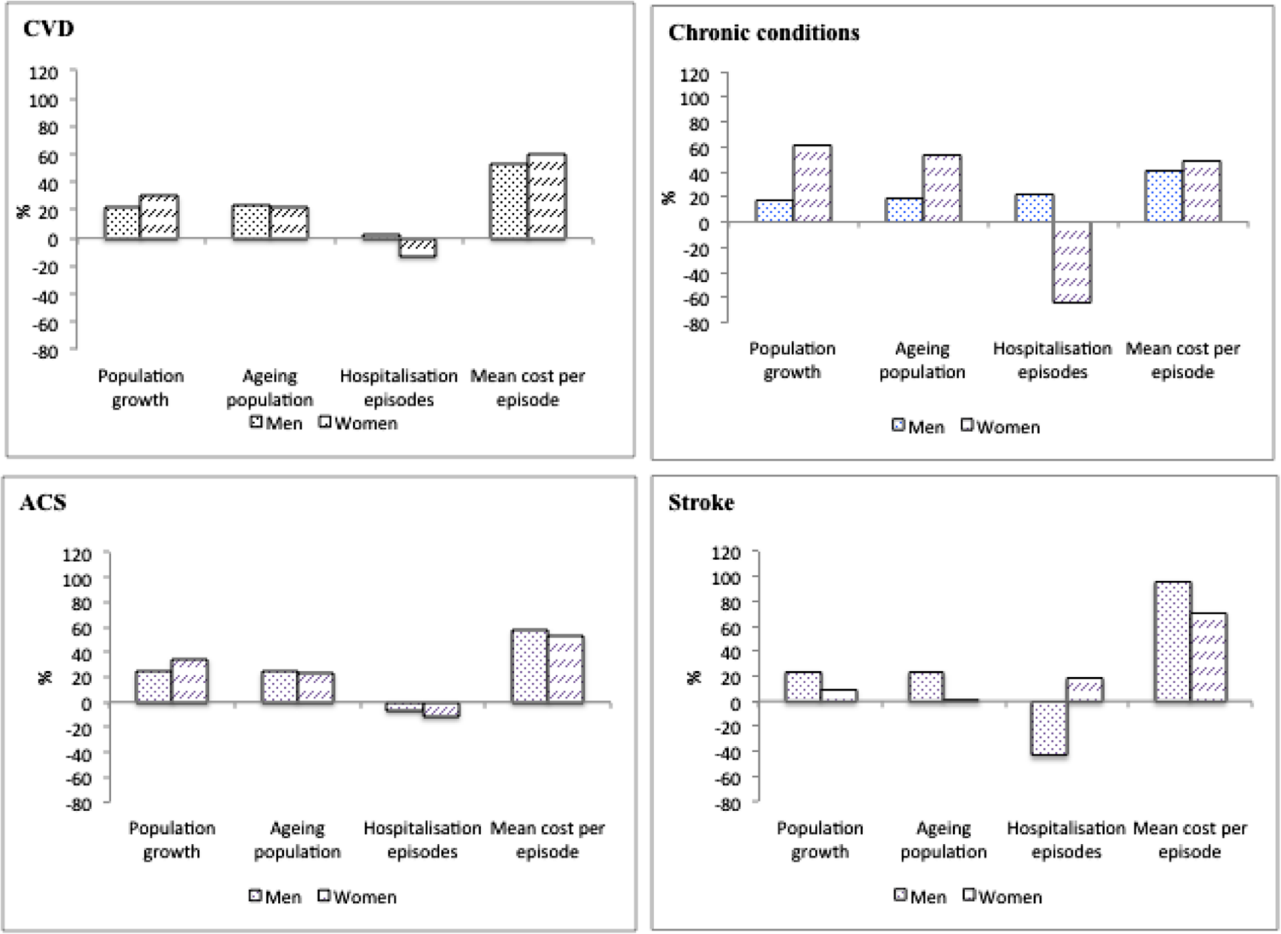
**Decomposition of increasing total costs of hospitalisation for CVD and sub-groups by components among men and women in 1993/94 to 2003/04.**

The results of the component decomposition analysis are presented in Figure 1. For overall CVD, the contribution of population ageing to the increase in the total cost of hospitalisation was 22.9% in men and 22.7% in women. If only total population had grown, but the age distribution remained constant as in 1993/94, the total costs of hospitalisation for CVD would have increased by 21.5% in men and 30.1% in women. More than half of the increase the total hospitalisation costs was attributable to the increase in average cost per episode. In contrast, if only the change in the number of episodes was considered, total cost would have reduced by 12.6% in women and increased by only 2.7% in men.

As shown in Figure 1, the contribution of the ageing population to the increase in the total costs of hospitalisation varied according to the subgroups, especially among women. For chronic conditions, ageing of the population accounted for over 50% of the rise in total costs among women, but only 23.5% in women with ACS and 1.7% in women with stroke. If only the change in the mean cost per episode was taken into account, this component would contribute to nearly 50% of the rise in the total costs of hospitalisation for chronic conditions and ACS in both men and women. In contrast, the change in the mean cost per episode accounted for 96.2% and 70.9% in men and women respectively of the increase in the total costs of hospitalisation for stroke.

The contribution of the ageing population to the rise in the total costs of hospitalisation distinctly differed between men and women (Figure 1). For example, the contribution of ageing to the rise of the total costs of hospitalisation was almost three times higher in women than men for chronic conditions, 53.9% compared with 19.1%. In contrast, the impact of the ageing population on the costs of hospitalisation for stroke was far greater in men than in women (23.6% vs. 1.7%). Thus, ageing had a higher impact on the rise of total cost for chronic conditions compared with ACS and stroke and the impact was different between men and women.

## Discussion

This study has examined the impact of ageing on the increase in hospitalisation costs for CVD and subgroups using the component decomposition method. The impact of ageing was separated out from other components including population growth, growth in the number of hospitalisations and the increase in mean cost per episode. The study showed that between 1993/94 and 2003/04 ageing contributed a considerable proportion to the increase in hospitalisation costs for CVD, 22.9% in men and 30% in women, although it was not the major component. The impact of ageing also varied across CVD conditions and was much higher for chronic conditions than acute conditions (ACS and stroke).

The findings of this study are consistent with other studies’ findings that ageing has an impact on healthcare expenditure but is not a major contributor [9,13,28]. However, in other studies the impact of ageing was found to be lower (less than 10%) [13,28]. The differences in the magnitude of impact could be due to differences in the type of expenditure. Other studies were conducted in the US and were based on Medicare fees [28], and in Korea using health insurance expenditure [13], and included a wide range of diseases. This study focused on CVD hospitalisation costs, which constitute the largest share of CVD costs in Australia, and ageing is a major risk factor for CVD [29,30]. The result of the study may imply that a higher impact of ageing population on CVD costs than general healthcare expenditure. The findings are also in agreement with other studies about the impact of ageing in Australia [6,7]. Although these other studies did not directly measure the magnitude of the change in the age structure on the hospitalisation costs for CVD, their results implied a potential burden of healthcare services due to ageing. Lynch, Holman and Moorin [7] found that the average age for first time hospitalisations declined over the period between 1980 and 2003, together with an increase in life expectancy. Thus, the life span living with chronic conditions increased, inducing higher demand and cost for healthcare.

Although the impact of ageing on healthcare expenditure has been measured in many studies, little is known about gender disparity [10,13,31]. Our results show a higher impact of ageing on hospitalisation costs for CVD in women than in men. This is explained by the finding that lifetime healthcare expenditure in women was higher than in men [32,33]. It is also a consequence of a higher life expectancy and higher prevalence of chronic disease among women compared with men [31].

Of interest is that the magnitude of the impact of ageing on hospitalisation costs varied widely between subgroups in women. Ageing was a main driver of the rise in the total cost of hospitalisation for chronic conditions, but not for ACS and stroke. For ACS and stroke, the main driver of the rise in total costs of hospitalisation was the growth in average cost per hospitalisation. This result is consistent with a cross sectional study conducted on stroke, which found that ageing had a relatively small impact on healthcare expenditure [34]. The strong effect of ageing on the cost of hospitalisations for chronic conditions may reflect the expansion of morbidity associated with chronic disease in Australia [7]. Coupled with this, the mortality rate for heart failure, a main component of chronic conditions, was found to have declined in patients aged 75 years and older between 1997 and 2003 [35]. In addition, the proportion of people aged 65 years and older increased while the younger age groups remained stable [3]. All these factors may help explain the higher impact of ageing on hospitalisation costs for chronic conditions. In Australia, the “baby boomers” population cohort, who was born in the 20-year period after World War II, would have been reaching middle age in this study period between 1990 and 2004. As these results only reflect the impact of ageing before the period of the baby boomer cohort reaching older age, it is likely that the impact of ageing on hospitalization costs for chronic conditions may accelerate in coming years.

### Strengths and limitations of this study

The study used a validated and well respected source of data and linkage mechanism which has been shown to produce accurate (99% accuracy) individual level linked data [18]. The data included all hospitalisation occurring in the state (both private public and private) providing highly generalizable population level information. Thus, bias due to selection or sampling error is not present in this study, leading to an increase in study power due to the comprehensive population coverage. For the decomposition of the increase in healthcare costs, although major components contributing to the increase in total costs of hospitalisation were taken into account, the components could have been be broken down into more detail. Average cost per hospitalisation, for example, can be separated into number and type of services used within each episode and cost per service. However, the available costing data in the Hospital Morbidity Data System were not sufficient to allow further decomposition. Since the focus of the study was to evaluate the contribution of the ageing component to CVD hospitalisation costs, this limitation did not affect our ability to fulfil this aim. Another limitation is that the time period for the analysis was 1993/94 to 2003/04. Since 2004, many changes in medical management and advances in diagnosis have taken place. Nevertheless, the findings of this study on the impact of ageing on the costs of hospitalisation for CVD and subgroups provides a useful basis for projecting corresponding costs in future time periods. Despite its limitations, the study has shown the extent to which ageing has impacted on costs of hospitalisation for CVD.

## Conclusions

This study has demonstrated that population ageing was a considerable factor, contributing to more than 20% of the rise in the total hospitalisation costs for CVD. The magnitude of the impact of ageing varied across CVD conditions, particularly among women. It was the main driver in the rise of total hospitalisation costs for chronic condition in women but only a minor factor for ACS and stroke. These results suggest that disease-specific factors should be considered when measuring the impact of ageing on healthcare expenditure and planning for healthcare budgets, as the magnitude of the impact was different between acute and chronic conditions. Additional studies using current data are needed to evaluate the impact of more recent technological/clinical advancement on hospitalisation costs of CVD.

## Additional file

Additional file 1
**Equations for decompose component analysis.**
