# Supplementary material for: Impact of population ageing on the costs of hospitalisations for cardiovascular disease: a population-based data linkage study
Source: BMC Health Serv Res. 2014 Nov 13;14:554. doi: 10.1186/s12913-014-0554-9 (PMC4236486; doi:10.1186/s12913-014-0554-9)
Supplement: Additional file 1 — Equations for decompose component analysis. [file 12913_2014_554_MOESM1_ESM.docx]

**Appendix 1: Equations for decompose component analysis**

The following steps and equations demonstrate how to measure the contribution of each component between 1993/94 and 2003/04, 0 and t denote the two time points, respectively. The following calculations were conducted separately for chronic conditions, ACS, stroke and overall CVD.

***Actual change in total costs***

The actual change in total hospitalization costs from 1993/94 to 2003/04 was attributable to population growth, ageing population, growth in number of episodes and change in cost per episode. It was a difference in total costs of hospitalisation for a disease (d) between two points of times 1993/94 and 2003/04, labelled as Cr^:^

*Cr = C0 - Ct (2)*

Where:

C0 is the total costs of hospitalisation for a disease d in 1993/94;

Ct is the total costs of hospitalisation for a disease d in 2003/04

***The proportion of change in total costs attributable to each component***

To capture the proportion of each component contributing to the difference in total cost of hospitalisation in the period, each component in initial year (1993/94) was moved to actual value in 2003/04 in sequence. As each component in the assumption was changed, the difference in total cost of hospitalisation between initial year (1993/94) and the assumed final year (2003/04) was attributable to whichever component was affected by the change in assumption. Details of the calculations are presented in following table.

| **The assumption total hospitalisation costs** | **The proportion of change in total costs attributable to each component** |
| --- | --- |
| **The first assumption:**  Only total population increased to actual values in 2003/04 while the age structure of the population, number of episodes per capita and cost per episode were kept constant at 1993/94 levels  The assumption total hospitalisation costs was labelled as C1  *C1 = ∑ (WA population A _j_ * number of episodes per capita _0,j_ * cost per episode_0j_)*  Where “*WA population A _j_*_”_ is the assumption population for age group j, calculated as following:  *WA population A _j_= Total WA population in 2003/04 * proportion of population in age group j in 1993/94* | - From the first assumption, the change in total costs attributable to **population growth (CPOP)** were calculated as following:   CPOP = $\frac{C1-C0}{Cr}$* 100  Where:  C0 is the total costs of hospitalisation for a disease d in 1993/94  Cr is a actual change in total costs of hospitalisation between two points of times 1993/94 and 2003/04 |
| **The second assumption:**  Both the total population and age structure of the population changed to values in 2003/04 while the number of episodes per capita and cost per episode were constant at 1993/94 levels  The assumption total hospitalisation costs was labelled as C2  *C2 = ∑(WA population _tj_ * number episodes per capita _0j_ * cost per episodes_0j_ )*  Where “WA population _tj”_ is the population in 2003/04 for age group j | - From the second assumption, the change in total costs attributable to the change in age structure of the WA population (CDEM1) was calculated as following:   CDEM1= $\frac{C2-C1}{Cr}$* 100  Where:  C1 is the first assumption total hospitalisation costs in 2003/04  Cr is a actual change in total costs of hospitalisation between two points of times 1993/94 and 2003/04 |
| **The third assumption:**  **T**he population in both number and age structure and total number of episodes changed to values in 2003/04 but the age distribution of episodes and cost per episode were unchanged at 1993/94 levels. The assumption total hospitalisation costs was labelled as C3  C3 *= ∑(WA population _tj_ * Number of episodes per capita A _j_ * cost per episode _0j_)*  Where “*Number of episodes per capita A _j_*_”_ is the assumption number of episodes per capital for age group j, calculated as following:  *Number of episodes per capita A_j_ = Assumption total number of episodes _j_* / *WA population _t,j_*  *Where: Assumption total number of episodes _j_ =Total number of episodes in 2003/04 * proportion of episodes in age group j in 1993/94* | - From the third assumption, the change in the total hospitalisation cost attributable to a change in the total number of episodes (CTNE) was calculated as following   CTNE= $\frac{C3-C2}{\mathrm{Cr}}$* 100  Where:  C2 is the second assumption total hospitalisation costs in 2003/04  Cr is a actual change in total costs of hospitalisation between two points of times 1993/94 and 2003/04 |
| **The fourth assumption:**  Both the WA population and the number of episodes per capita were as in 2003/04 and only the cost per episode was constant at 1993/94 levels.  The assumption total hospitalisation costs was labelled as C4  C4 *= ∑(WA population _tj_ * number of episodes per capita_tj_ * cost per episode _0j_)* | From the fourth assumption:   - Change in total cost attributable to a change in age distribution of hospitalisations (CDEM2) was calculated as following:   CDEM2= $\frac{C4-C3}{Cr}$* 100  Where:  C3 is the third assumption total hospitalisation costs in 2003/04  Cr is a actual change in total costs of hospitalisation between two points of times 1993/94 and 2003/04   - And the share of the change in total hospitalisation costs due to the change in cost per episode (CCPE) was also calculated as   CCPE= $\frac{Ct-C4}{Cr}$* 100  Where:  Ct is the actual total costs of hospitalisation for in 2003/04  Cr is a actual change in total costs of hospitalisation between two points of times 1993/94 and 2003/04 |
| Change in total cost attributable to ageing = CDEM1 + CDEM2 | |
